# Deep-branching ANME-1c archaea grow at the upper temperature limit of anaerobic oxidation of methane

**DOI:** 10.3389/fmicb.2022.988871

**Published:** 2022-09-23

**Authors:** David Benito Merino, Hanna Zehnle, Andreas Teske, Gunter Wegener

**Affiliations:** ^1^Max Planck Institute for Marine Microbiology, Bremen, Germany; ^2^Faculty of Geosciences, University of Bremen, Bremen, Germany; ^3^MARUM, Center for Marine Environmental Sciences, University of Bremen, Bremen, Germany; ^4^Department of Earth, Marine and Environmental Sciences, University of North Carolina at Chapel Hill, Chapel Hill, NC, United States

**Keywords:** anaerobic oxidation of methane, ANME-1, archaea, deep sea, hydrothermal vents

## Abstract

In seafloor sediments, the anaerobic oxidation of methane (AOM) consumes most of the methane formed in anoxic layers, preventing this greenhouse gas from reaching the water column and finally the atmosphere. AOM is performed by syntrophic consortia of specific anaerobic methane-oxidizing archaea (ANME) and sulfate-reducing bacteria (SRB). Cultures with diverse AOM partners exist at temperatures between 12°C and 60°C. Here, from hydrothermally heated sediments of the Guaymas Basin, we cultured deep-branching ANME-1c that grow in syntrophic consortia with *Thermodesulfobacteria* at 70°C. Like all ANME, ANME-1c oxidize methane using the methanogenesis pathway in reverse. As an uncommon feature, ANME-1c encode a nickel-iron hydrogenase. This hydrogenase has low expression during AOM and the partner *Thermodesulfobacteria* lack hydrogen-consuming hydrogenases. Therefore, it is unlikely that the partners exchange hydrogen during AOM. ANME-1c also does not consume hydrogen for methane formation, disputing a recent hypothesis on facultative methanogenesis. We hypothesize that the ANME-1c hydrogenase might have been present in the common ancestor of ANME-1 but lost its central metabolic function in ANME-1c archaea. For potential direct interspecies electron transfer (DIET), both partners encode and express genes coding for extracellular appendages and multiheme cytochromes. *Thermodesulfobacteria* encode and express an extracellular pentaheme cytochrome with high similarity to cytochromes of other syntrophic sulfate-reducing partner bacteria. ANME-1c might associate specifically to *Thermodesulfobacteria,* but their co-occurrence is so far only documented for heated sediments of the Gulf of California. However, in the deep seafloor, sulfate–methane interphases appear at temperatures up to 80°C, suggesting these as potential habitats for the partnership of ANME-1c and *Thermodesulfobacteria*.

## Introduction

In anoxic deep-sea sediments, the greenhouse gas methane is produced abiotically by thermocatalytic decay of buried organic matter or biotically by methanogens (Whiticar, 1999). Anaerobic oxidation of methane (AOM) mitigates the flux of methane to the water column and eventually to the atmosphere by consuming 90% of the methane produced in the deep sediments (Hinrichs and Boetius, 2002; Reeburgh, 2007; Regnier et al., 2011). In marine sediments, AOM primarily couples to sulfate reduction in a 1:1 stoichiometry:


(1) $${CH}_{4}+{\mathrm{SO}}_{4}^{2-}\to {HS}^{-}+{HCO}_{3}^{-}+H_{2}O\text{.}$$

AOM is mediated by anaerobic methanotrophic archaea (ANME) that oxidize methane to CO_2_ by reversing the methanogenesis pathway (Hallam et al., 2004; Meyerdierks et al., 2010; Wang et al., 2013). ANME do not encode respiratory pathways, but they pass the reducing equivalents liberated during AOM to sulfate-reducing partner bacteria (SRB), forming characteristic consortia (Boetius et al., 2000; Michaelis et al., 2002; Orphan et al., 2002; McGlynn et al., 2015; Wegener et al., 2015). The nature of this syntrophic association and the mechanisms involved in the transfer of reducing equivalents from ANME toward SRB are not completely resolved at the molecular level. Originally, it was proposed that the archaeal partners produce molecular hydrogen that is consumed by the bacterial partners (Hoehler et al., 1994). However, most ANME do not code for hydrogenases (Chadwick et al., 2022). Previous studies support the hypothesis of direct interspecies electron transfer (DIET) involving multiheme cytochromes and pilus proteins (Meyerdierks et al., 2010; McGlynn et al., 2015; Wegener et al., 2015). The partner SRBs use the AOM-derived electrons for anaerobic respiration with sulfate as final electron acceptor (Boetius et al., 2000; Wegener et al., 2015; Laso-Pérez et al., 2016). The limited energy yield of sulfate-dependent AOM [equation (1), ΔG°′ = −16.67 kJ mol^−1^ at standard conditions and ΔG = −20 to −40 kJ mol^−1^ in marine AOM habitats] needs to be shared between ANME and their syntrophic partner SRB (Thauer, 2011).

ANME inhabit a variety of marine habitats including cold seeps (Boetius et al., 2000; Orphan et al., 2001), mud volcanoes (Niemann et al., 2006), gas hydrates (Lanoil et al., 2001; Orcutt et al., 2004), hydrothermal vents (Inagaki et al., 2006; Biddle et al., 2012) and deep subsurface sediments (Roussel et al., 2008). ANME are polyphyletic and fall into three distinct phylogenetic groups (ANME-1, ANME-2, and ANME-3). ANME-3 often dominate AOM at mud volcanoes, where they form consortia with *Desulfobulbus*-related bacteria (Niemann et al., 2006; Lösekann et al., 2007). Cultivation attempts of ANME-3 have not been successful so far. ANME-2 are globally distributed in a variety of benthic habitats and are typically found associated with *Desulfosarcina*/*Desulfococcus* bacteria (DSS, Seep-SRB1 and Seep-SRB2 clades; Knittel et al., 2003, 2005; Boetius and Knittel, 2010). ANME-2 are dominant at cold seeps with high methane fluxes and temperatures below 20°C (Knittel et al., 2005). Cultivation attempts at temperatures ≤ 20°C resulted in the enrichment of ANME-2c (Holler et al., 2009; Wegener et al., 2016).

ANME-1 prevail in most deep sulfate–methane transition zones (SMTZs; Ishii et al., 2004; Niemann et al., 2005; Treude et al., 2005), in hydrothermally heated sediments in the Guaymas Basin (Teske et al., 2002; Schouten et al., 2003; Ruff et al., 2015; Dombrowski et al., 2018), and in the Auka vent field, in the Pescadero Basin (Gulf of California; Speth et al., 2022). Meso- and thermo-philic AOM cultures have been obtained from Guaymas Basin sediments at 37°C, 50°C, and 60°C (Holler et al., 2011; Wegener et al., 2016). These cultures consisted of ANME-1a and HotSeep-1 (*Ca.* Desulfofervidus) as partner bacteria. *Ca.* Desulfofervidus sequences are also found *in situ* at these sites (McKay, 2014; Dowell et al., 2016).

Previous short-term incubations revealed AOM activity at temperatures up to 75°C or 85°C, but the microorganisms performing AOM under these conditions were not assessed (Kallmeyer and Boetius, 2004; Holler et al., 2011; Adams et al., 2013). Here, we obtained an active AOM culture at 70°C (AOM70) from Guaymas Basin hydrothermal sediments consisting of a previously uncultured ANME-1 subgroup (Teske et al., 2002) and an apparently obligate syntrophic *Thermodesulfobacterium* partner. We describe their function and interaction based on physiological experiments and molecular data.

## Materials and methods

### Sediment collection and enrichment culture setup

Sediment push cores from gas-rich hydrothermal vents of the Guaymas Basin (Gulf of California) were collected by the submersible *Alvin* at 2013 m depth during RV *Atlantis* cruise AT42-05 (November 2018). Sediments for this AOM enrichment came from cores 4,991–13 and 4,991–14 in the Cathedral Hill area (27°00.6848′ N, 111° 24.2708′ W) collected on 17 November 2018, in an area covered by dense orange-white *Beggiatoaceae* mats, where temperatures at 50 cm depth reached at least 80°C. On board sediment samples were transferred to glass bottles sealed with butyl rubber stoppers, the headspace was exchanged to argon. Sediments were stored at 4°C until further processing. Sediment slurries were prepared following protocols previously described (Laso-Pérez et al., 2018). Anoxic sediments were mixed with sulfate-reducer medium (Widdel and Bak, 1992) in a 1/10 ratio (v/v) in serum vials sealed with rubber stoppers. The headspace of the serum vials was replaced with 2 atm methane:CO_2_ (90:10). The dry weight of the original slurries was 60 g L^−1^. The slurries were incubated at 70°C in the dark. Methane-dependent sulfide production was measured with the copper sulfate assay (Cord-Ruwisch, 1985). Incubations with methane-dependent sulfide productions at 70°C are referred to as AOM70 culture. These cultures were diluted 1/5 with new medium when sulfide levels reached > 10 mM. Cultures were virtually sediment-free after four dilutions.

### DNA extraction and long-read sequencing

DNA samples for long-read sequencing were prepared according to previous protocols with few modifications (Zhou et al., 1996; Hahn et al., 2020). In short, 50 ml culture were collected in a Falcon tube and biomass was pelleted by centrifugation at RT (4,000 rpm for 20 min). After removing the supernatant, 800 μl extraction buffer was added (100 mM tris–HCl, 100 mM sodium EDTA, 100 mM sodium phosphate, 1.5 M NaCl, 1% CTAB, pH 8). For physical lysis of cell envelopes, the pellet suspension was frozen twice in liquid nitrogen and thawed in a water bath at 65°C. For enzymatic lysis, 1,000 μl extraction buffer with 60 μl proteinase K (20 mg mL^−1^) was used at 37°C for 1.5 h with constant shaking. Chemical lysis was done with 300 μl 20% SDS at 65°C for 2 h. Cell debris was pelleted again by centrifugation at RT (13,000 × *g* for 20 min). The clear supernatant was transferred to a new tube and 2 ml of chloroform-isoamyl alcohol (16:1, v:v) were added. The samples were mixed by inverting the tubes and centrifuged at RT (13,000× *g* for 20 min). The aqueous phase was transferred to a new tube and mixed with 0.6 volumes isopropanol. DNA was precipitated overnight at −20°C. After precipitation, DNA was re-dissolved at 65°C in a water bath for 5 min and samples were centrifuged at RT (13,000× *g* for 40 min). Supernatant was removed and the pellet was washed with ice-cold 80% ethanol. Samples were centrifuged at 13,000× *g* for 10 min and the ethanol was removed. Dried pellets were resuspended in 100 μl PCR-grade water. Long-read (>10 kb) genomic DNA was sequenced on a Sequel IIe (Pacific Biosciences) at the Max Planck Genome Centre in Cologne. Read length distributions and abundances are compiled in Supplementary Table 1.

### RNA extraction and short-read shotgun sequencing

Triplicates of 30 ml culture were filtered onto 0.2 μl polycarbonate filters under gentle vacuum. Filters were soaked immediately with RNAlater (Invitrogen) preheated at 70°C for 10–15 min. RNAlater was removed by filtration and the filters were stored at −20°C until further processing. For RNA extraction, ¼ of a filter was put into a bead-beating tube (Lysing Matrix E, MPBio) together with 600 μl RNA lysis buffer (Quick-RNA MiniPrep kit, Zymo Research). Tubes were vortexed at maximum speed for 20 min. Biomass was pelleted by centrifugation at RT (10,000× *g* for 5 min). The supernatant was collected and RNA was extracted with the Quick-RNA MiniPrep kit (Zymo Research) including a DNA digestion step with DNase I. Total RNA libraries were sequenced in an Illumina HiSeq2500 machine at the Max Planck Genome Centre (Cologne, Germany). We obtained 4 Mio 2 × 250 bp paired-end reads.

### Metagenome and metatranscriptome analysis

Metagenomic long-reads were assembled using Flye v. 2.9 (Kolmogorov et al., 2020). Shotgun metatranscriptomic short reads were quality trimmed using BBduk from the BBtools package v. 38.87^1^ with the parameters minlength = 50 mink = 6 hdist = 1 qtrim = r trimq = 20. Metagenomic reads were mapped to the general assembly. Long reads were mapped using minimap2 v. 2.21 (Li, 2018) with default parameters. Open reading frames in metagenomic contigs were predicted with prodigal v. 2.6.3 (Hyatt et al., 2010) and genes were annotated with PFAMs, TIGRFAMs, COGs, KEGGs, and RNAmmer (Kanehisa and Goto, 2000; Haft et al., 2001; Lagesen et al., 2007; Galperin et al., 2015; Mistry et al., 2021). CXXCH motifs in putative multiheme cytochromes were searched with a custom script.^2^ Predicted hydrogenase sequences were classified into subgroups with the hydrogenase database (HydDB; Søndergaard et al., 2016). Subcellular localization of heme-containing proteins and hydrogenases was predicted with PSORTb 3.0 (Yu et al., 2010). Metagenomic binning based on differential coverage across metagenomic samples was done with maxbin v 2.2.7 (Wu et al., 2016). Bins were manually refined in anvi’o v. 6 (Eren et al., 2015, 2020) by removing contigs with low coverage from high-coverage bins.

Triplicate metatranscriptomes were mapped to curated bins using bowtie 2 (Langmead and Salzberg, 2012). The rRNA and tRNA gene sequences were removed before calculating gene expression levels. Center-log ratio (CLR) values for relative gene expression were calculated according to the formula:


(2) $$CLR_{i}=\log_{2}\frac{x_{i}}{L_{i}\sqrt[{n}]{x_{1}\times x_{2}\times \ldots \times x_{n}}}$$

where $x_{i}$ are the reads mapped to a specific gene and $L_{i}$is the length of the gene in kbp. A 0.5 factor was added to read-mapping values to avoid zero values.

To analyze the similarity of cytochrome-like proteins in ANME-1 and sulfate-reducing bacteria, amino acid sequences from sulfate-reducing partner bacteria genomes were downloaded from NCBI (Krukenberg et al., 2016, 2018). BLAST databases were created from the cytochrome sequences in the reference SRB genomes using makeblastdb (BLAST v. 2.10.1; Altschul et al., 1990). Cytochrome-like proteins in AOM70 cultures were queried against the custom database with BLASTp v 2.5.

### Community composition and phylogenetic analyses based on the 16S rRNA gene

16S rRNA genes from long-read metagenomic assemblies were extracted with Metaxa2 (Bengtsson-Palme et al., 2015). Full-length 16S rRNA gene sequences were aligned to the SILVA database release 138.1 using the SINA aligner within the ARB software (Ludwig et al., 2004; Pruesse et al., 2012; Quast et al., 2013). Long reads were mapped against 16S rRNA genes using minimap2 (Li, 2018). Shotgun metatranscriptomic reads were aligned to 16S rRNA gene using bowtie2 (Langmead and Salzberg, 2012). Maximum-likelihood 16S rRNA phylogenetic trees with selected ANME or *Thermodesulfobacteria* sequences were calculated using RAxML with 1,000 bootstraps and a 50% frequency base filter (Stamatakis, 2014).

### Phylogenomic and phylogenetic analyses

Archaea and Bacteria genomes were downloaded from public databases (Supplementary Table 3). For ANME-1 phylogenomic analysis, the genomes were annotated with HMMs of 38 conserved archaeal marker genes (Supplementary Table 3; Darling et al., 2014). For *Thermodesulfobacteria*, the genomes were annotated with HMMs of 71 conserved bacterial marker genes (Rinke et al., 2013). The amino acid sequences of each set were aligned and concatenated using MUSCLE (Edgar, 2004). Maximum likelihood phylogenomic trees were calculated with IQTree using the –test option to estimate the best substitution model for each protein in the partition file and using 100 bootstraps (Chernomor et al., 2016; Kalyaanamoorthy et al., 2017; Minh et al., 2020). Reference hydrogenase sequences (Supplementary Table 5) were downloaded from the hydrogenase database (HydDB; Søndergaard et al., 2016). ANME-1 hydrogenases and reference hydrogenases were aligned with muscle (Edgar, 2004). Maximum likelihood phylogenetic trees of the alignment were calculated with IQTree with 100 bootstraps (Chernomor et al., 2016; Kalyaanamoorthy et al., 2017; Minh et al., 2020). Trees were visualized and edited on the Interactive Tree Of Life (iTOL) online server (Letunic and Bork, 2011).

### Catalyzed reporter deposition-fluorescent *in situ* hybridization

To prepare catalyzed reporter deposition-fluorescent *in situ* hybridization (CARD-FISH) samples, 5 ml culture were fixed at 1% formaldehyde concentration over night at 4°C. Fixed samples were sonicated (15 s, 30% power, 20% cycle) to detach cells from sediment particles to detach larger aggregates. Samples were then filtered onto 0.2 μm polycarbonate filters and fixed with 0.2% low-melting agarose before CARD-FISH. Samples were stored at −20°C until further processing. CARD-FISH was performed as described previously (Pernthaler et al., 2002). In short, endogenous peroxidases were inactivated with a solution of 0.15% H_2_O_2_ in methanol for 30 min at RT. Cell walls were permeabilized with lysozyme (Sigma Aldrich, 10 mg mL^−1^ lysozyme in 50 mM EDTA, 100 mM Tris–HCl; 60 min incubation at 37°C), proteinase K (15 μg mL^−1^ proteinase K in 50 mM EDTA, 100 mM Tris–HCl, 500 mM NaCl; 10 min incubation at RT) and HCl (0.1 M HCl; 1 min incubation at RT). Horseradish peroxidase-labeled probes were diluted in hybridization buffer at the adequate formamide concentration for each probe (Supplementary Table 2). Probes were hybridized at 46°C for 2 h. Signal amplification with fluorescent tyramides was done for 45 min at 46°C. For double hybridizations, peroxidases of the prior hybridization step were inactivated by incubating the filter in 0.30% H_2_O_2_ in methanol for 30 min at RT.

### Quantification of methane and hydrogen in AOM cultures

For cultures under AOM conditions, hydrogen formation in the headspace was measured by gas chromatography coupled to reducing compound photometry (RCP, Peak Performer 1 RCP, Peak Laboratories). For cultures under methanogenic conditions, methane formation in the headspace was measured *via* gas chromatography and flame ion detection (GC-FID, Focus GC, Thermo).

## Results and discussion

### AOM enrichment cultures at 70°C

**Figure 1:**
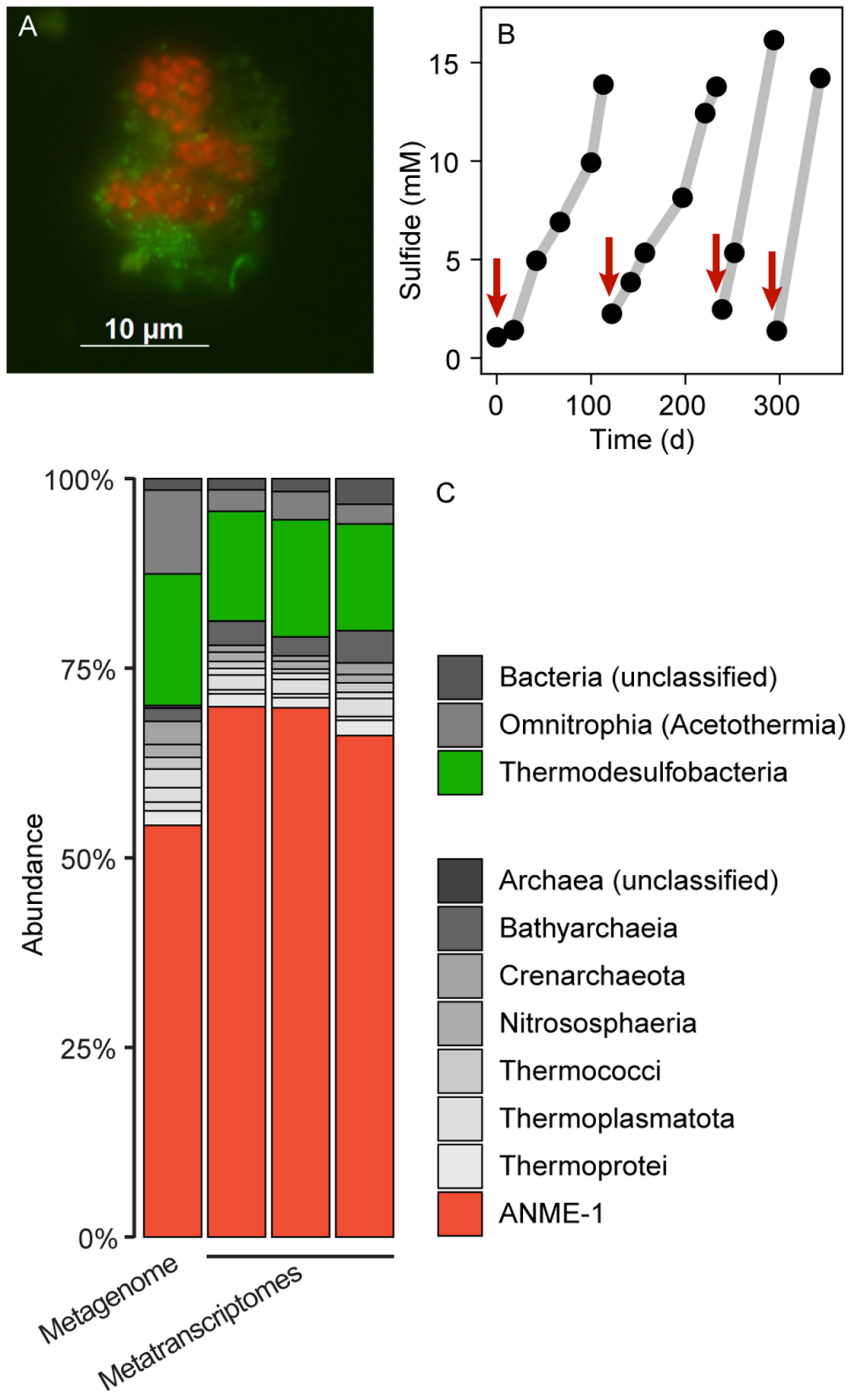
Microbial composition and growth of thermophilic AOM cultures. **(A)** CARD-FISH on AOM aggregates with the probes EUB388 I-III (green) and ANME-1-389 (red). Bacteria (green) and archaea (red) form shell-type consortia. **(B)** Methane-dependent sulfide production in AOM cultures. The red arrows indicate when culture medium was replaced. **(C)** 16S rRNA gene relative abundance in long-read metagenomic reads and shotgun metatranscriptomic reads (triplicate metatranscriptomes). The enrichment is dominated by ANME-1 and sulfate reducers of the class Thermodesulfobacteria. Other bacteria and archaea such as Acetothermia and Bathyarchaeia are side community members from original sediment samples.

A slurry produced from hydrothermally-heated sediments from the Guaymas Basin and sulfate-reducer medium was supplemented with a methane:CO_2_ headspace and incubated at 70°C. These incubations showed methane-dependent sulfate reduction, as measured by an increase of sulfide in the medium (Figure 1B; Supplementary Figure 1). These incubations produced sulfide 12 to 15 mM sulfide within about 100 days. The slurries were diluted 1/10 (v/v) in fresh SRB medium, and a fresh methane:CO_2_ headspace was added and the incubation was proceeded. After four additional incubation and dilution steps, the produced AOM70 cultures were virtually sediment-free, contained microbial aggregates visible with the naked eye and produced approximately. 100 μmol sulfide L^–1^ d^–1^. To our knowledge, this is the first long-term cultivation of AOM-performing microorganisms above 60°C. The culture showed strongly decreased sulfide production at 60°C. It tolerated a transfer to 75°C, but became inactive at 80°C, confirming prior results on the upper-temperature limit of AOM made in short-term incubations with Guaymas Basin sediments (Holler et al., 2011; McKay et al., 2016).

### Thermophilic AOM community at 70°C

To resolve the microbial community composition of the AOM70 culture, we obtained a long-read metagenome and triplicate short-read metatranscriptomes. The community consisted mainly of ANME-1 (~50% relative abundance of mapped reads) and *Thermodesulfobacteria* (~20% relative abundance) based on 16S rRNA gene fragments recruited from the metagenome (Figure 1C) that were rare in the original sediment samples (Supplementary Figure 2). Metatranscriptomic samples were also dominated by ANME-1 (~70% relative abundance) and *Thermodesulfobacteria* (~15% relative abundance), forming the active AOM community at 70°C. Both the metagenome and metatranscriptome revealed noticeable populations of Bathyarchaeota and Acetothermia (<10% relative abundance of mapped reads). Yet, we were not able to reconstruct MAGs of these organisms; hence, their potential functions are unknown. Previous studies suggest that these organisms ferment or oxidize biomolecules produced by the AOM community (Kellermann et al., 2012; Dombrowski et al., 2017; Hao et al., 2018; Zhu et al., 2022).

After long-read metagenome assembly and binning, we obtained two high-quality MAGs of the two members of the AOM consortium (Table 1). The *Thermodesulfobacterium* MAG has a size of 1.7 Mbp and GC content of 29%. The bin is almost complete (98.6%) and has no contamination based on the presence of 104 bacterial single-copy marker genes (CheckM; Parks et al., 2015). The ANME-1 bin has a size of 1.5 Mbp and a GC content of 47.8%. The bin is 90.8% complete and has contamination of 7.9% based on 149 archaeal marker genes (CheckM; Parks et al., 2015).

**Table 1:** Metagenome-assembled genomes retrieved from AOM70 enrichment cultures.

	*Ca.* Thermodesulfobacterium	ANME-1c (*Ca.* Methanophagales)
No. of contigs	4	16
Genome size	1.702 Mbp	1.493 Mbp
L50/N50	2/808,565 bp	5/109,767 bp
GC content	29.0%	47.8%
Completeness^*^	98.6%	90.8%
Contamination^*^	<1%	7.89%

* Completeness and contamination were calculated with CheckM.

We attempted to visualize the enriched ANME-1 using a previously established probe targeting the whole ANME-1 clade (ANME-1-350, Supplementary Table 2; Boetius et al., 2000). *In situ* hybridization with the ANME-1-350 failed, because the probe has two mismatches with the 16S rRNA sequence of the enriched ANME-1. The 16S rRNA gene of this ANME-1 belong to a clade ancestral to all ANME-1a/b, namely ANME-1c (Supplementary Figure 4 and Discussion below; Laso-Pérez et al., 2022). A newly developed ANME-1-389 probe specifically binds to ANME-1c cells. Because the probes available for partner SRB do not target the 16S rRNA sequence of *Thermodesulfobacteria*, we designed three candidate probes to target this clade (Supplementary Table 2). Unfortunately, none of these probes hybridized the 16S rRNA of this organism after various CARD-FISH attempts (Supplementary Table 2). *Thermodesulfobacteria* are likely the partner bacteria of ANME-1c during AOM at 70°C based on the abundance of bacterial cells and their gene content (see Discussion below). Furthermore, all genes coding for dissimilatory sulfate reductase (*dsr*) in the metagenome belong to the *Thermodesulfobacterium* MAG. Double hybridization with the ANME-1c and the general bacterial probes (Supplementary Table 2) revealed a dominance of “shell-type” aggregates consisting of ANME-1c and partner bacteria (Figure 1A; Supplementary Figure 3). These consortia consist of clumps of ANME-1c cells, surrounded by smaller rod-shaped bacterial cells. These shell-type aggregates differ from the predominantly mixed-type aggregates of moderately thermophilic consortia growing at 50°C–60°C (Holler et al., 2011; Wegener et al., 2015). A shell-type growth morphology is often observed in cold-adapted ANME (Knittel et al., 2005). The reason for the different association types is unknown.

### Phylogeny of deep-branching ANME-1c

**Figure 2:**
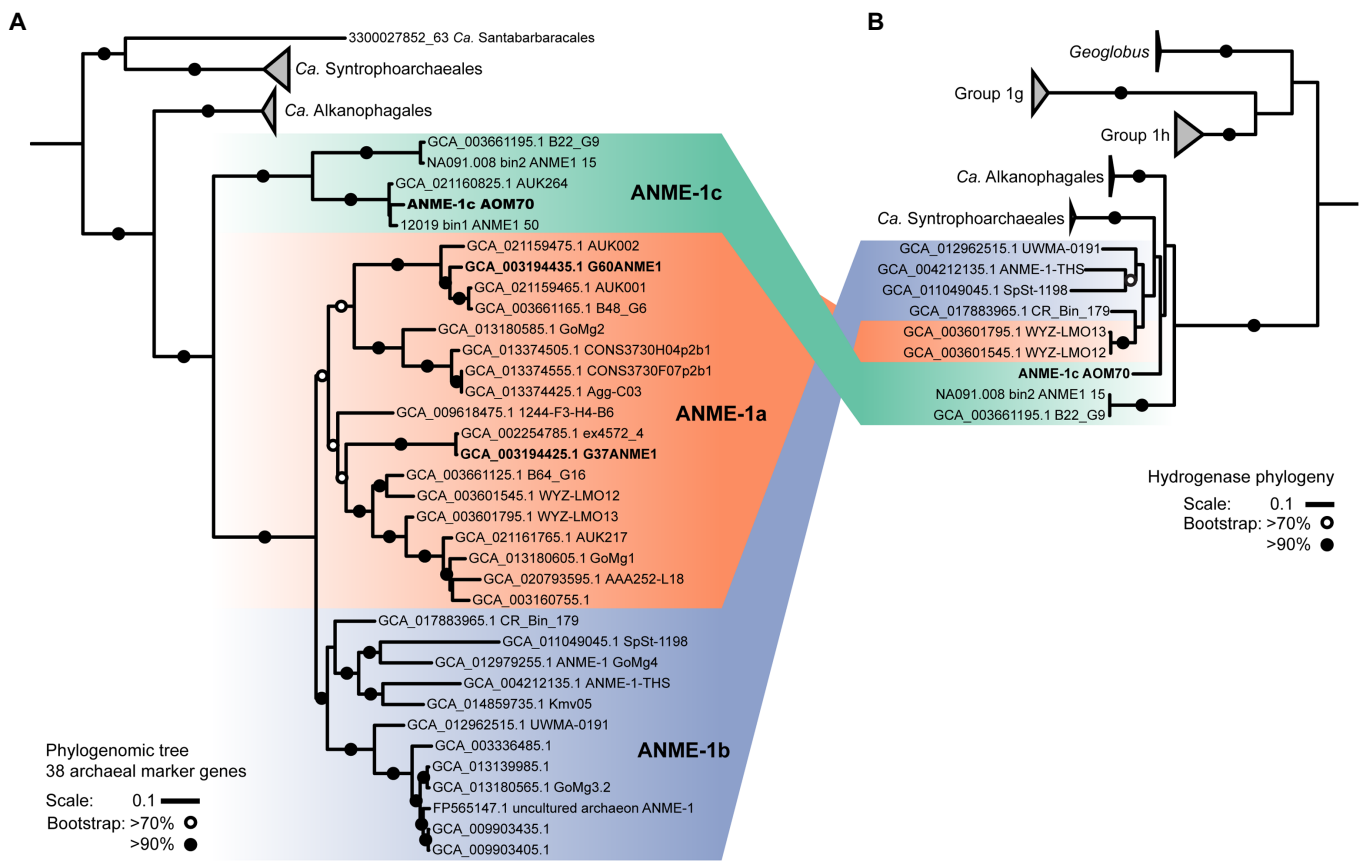
ANME-1 phylogenomic tree and hydrogenase phylogeny. **(A)** Phylogeny of ANME-1 order (*Ca.* Methanophagales) with related *Ca.* Alkanophagales, *Ca.* Syntrophoarchaeales, and *Ca.* Santabarbaracales. Maximum likelihood phylogenomic tree based on an alignment of 38 archaeal conserved genes from 55 genomes (Supplementary Table 3). *Geoglobus* sequences were the outgroup to set the tree root (not shown). **(B)** Hydrogenase phylogeny. ANME-1, *Ca.* Alkanophagales and *Ca.* Syntrophoarchaeales hydrogenases are located at the base of groups 1 g and 1 h of NiFe hydrogenases. A complete hydrogenase tree is shown in Supplementary Figure 11. MAGs from cultured ANME are depicted in bold. Shading in both trees indicates the three subdivisions of the ANME-1: ANME-1c, ANME-1a and ANME-1b. ANME-1 AOM70 is the genome discussed in the main text. Scales indicate nucleotide substitution per site. Bootstrap support is based on 100 iterations above 70% and above 90%.

On the basis of whole genome comparison, the ANME-1 population detected in the AOM70 culture falls into the recently named ANME-1c clade (Figure 2A; Laso-Pérez et al., 2022; Speth et al., 2022).The ANME-1c group is basal to its sister groups ANME-1a and ANME-1b within the order ANME-1 (*Ca.* Methanophagales). The 16S rRNA gene phylogenetic tree supports this phylogenetic placement (Supplementary Figure 4). ANME-1c belong to the class Syntrophoarchaeia with the ANME-1, *Ca.* Syntrophoarchaeales and *Ca.* Alkanophagales. Considering an average nucleotide identity (ANI) of <83% for distinct species and >95% for the same species (Jain et al., 2018) the ANME-1c clade consists of two distinct species clusters (Supplementary Figure 5). The ANME-1c MAG from the AOM70 culture belongs to the cluster of *Ca.* Methanoxibalbensis ujae from Pescadero Basin (Laso-Pérez et al., 2022). ANME-1c 16S rRNA gene sequences have been detected in hydrothermal sediments of the Guaymas Basin and the Juan de Fuca Ridge (Supplementary Figure 4; Teske et al., 2002; Merkel et al., 2013; McKay et al., 2016), and a MAG of the ANME-1c clade (accessions: SAMN09215218, GCA_003661195.1) was derived from Guaymas Basin hydrothermal sediments (Dombrowski et al., 2018). ANME-1c are also present in rock samples from hydrothermal fields in Pescadero Basin (Gulf of California; Speth et al., 2022). The ANME-1c clade was originally named “ANME-1b” by Teske and coworkers to differentiate this lineage from previously described cold-seep ANME-1 (Teske et al., 2002) and later renamed to ANME-1Guaymas because it was predominantly recovered from Guaymas Basin (Biddle et al., 2012; Merkel et al., 2013; Dowell et al., 2016). These sequences originate from sediment cores with sulfate-reducing activity at temperatures between 65°C and 90°C, showing that these archaea are likely all thermophiles (Biddle et al., 2012). Furthermore, the high GC content (>60%) of ANME-1c 16S rRNA genes indicates that these archaea might have temperature optima in the upper range of thermophily above 70°C (Merkel et al., 2013).

### Genomic and metabolic features of ANME-1c

**Figure 3:**
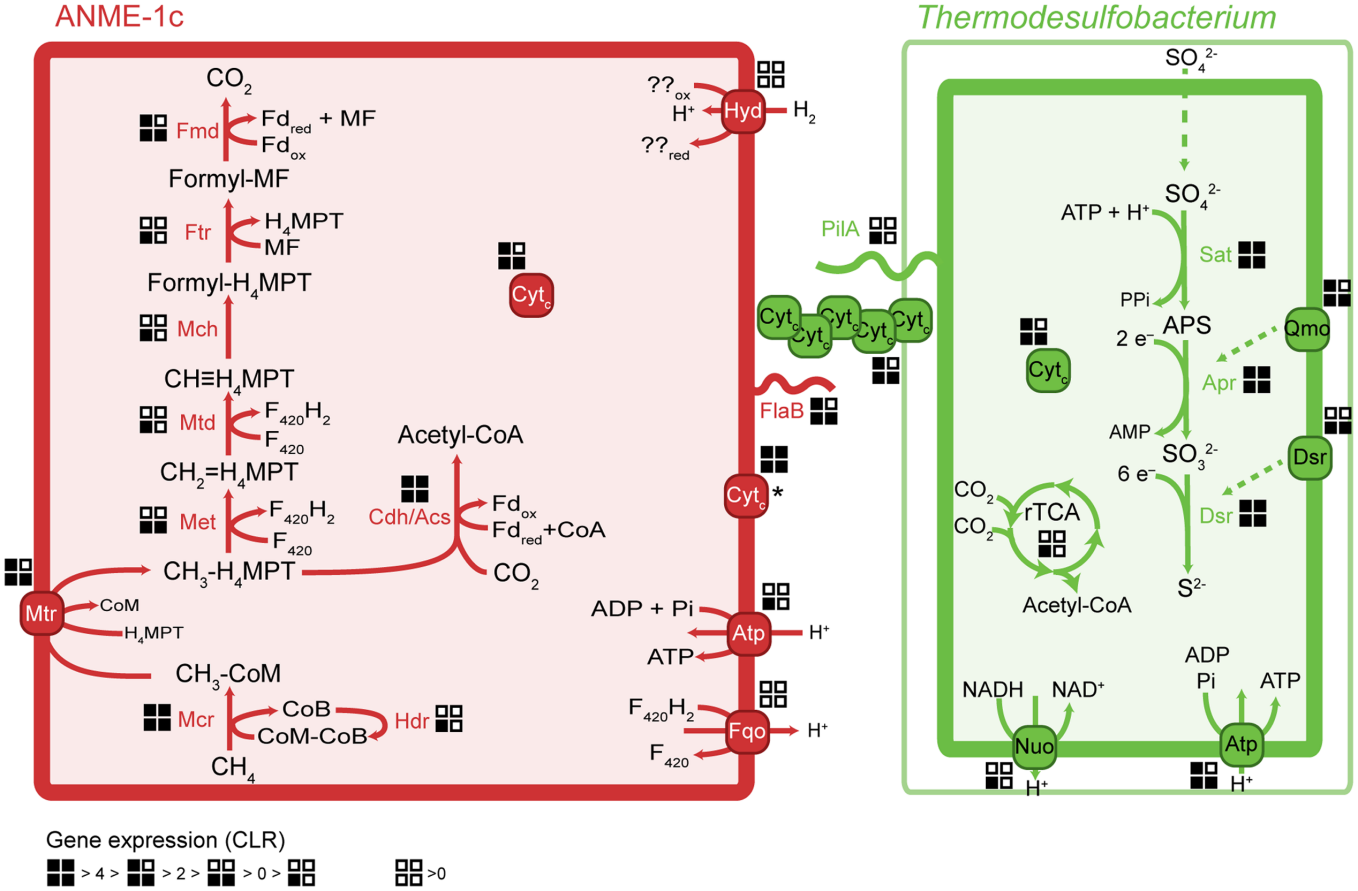
Key metabolic pathways in ANME-1c and *Ca.* Thermodesulfobacterium torris and metatranscriptomic expression during AOM. Gene expression values were normalized to centered-log ratios (CLR). A CLR value of 0 represents the mean expression of all genes in a genome. The asterisk next to the ANME-1c cytochrome indicates unknown cell localization. H_4_MPT, tetrahydromethanopterin; MF, methanofuran; Fd, ferredoxin; Mcr, methyl-coenzyme M reductase; Mtr, tetrahydromethanopterin S-methyltransferase; Met, 5,10-methylenetetrahydrofolate reductase; Mtd, methylenetetrahydromethanopterin dehydrogenase; Mch, methenyltetrahydromethanopterin cyclohydrolase; Ftr, formylmethanofuran-tetrahydromethanopterin formyltransferase; Fmd, formylmethanofuran dehydrogenase; Cdh/Acs, CO dehydrogenase/acetyl-coenzyme A synthase complex; rTCA, reductive tricarboxylic acid cycle; Sat, sulfate adenylyltransferase; Apr, adenylylsulfate reductase; Dsr, dissimilatory sulfate reductase; Fqo, ferredoxin: quinone oxidoreductase; Atp, ATP synthase; Hyd, hydrogenase; Qmo, quinone-modifying oxidoreductase; Nuo, NADH:ubiquinone oxidoreductase; Cyt_c_, multiheme cytochrome *c*-like protein; PilA, bacterial pilus protein; FlaB, archaeal flagellum protein (archaellum).

ANME-1c codes for a complete methanogenesis pathway including a canonical methane-active Mcr (Figure 3). The *mcr*ABC genes in ANME-1c have the highest expression (CLR > 7) among all genes in the dataset. This high expression of *mcr* confirms previous transcriptomic work in ANME (Haroon et al., 2013; Krukenberg et al., 2018). The activation of methane is the rate-limiting step of AOM, and ANME would promote this reaction by producing large amounts of Mcr (Scheller et al., 2010; Thauer, 2011). Similar to other ANME-1 archaea, ANME-1c does not encode a *N*^5^,*N*^10^-methylene-H_4_MPT reductase (*mer*). This gene might be substituted by a 5,10-methylenetetrahydrofolate reductase (*met;* Stokke et al., 2012; Krukenberg et al., 2018). The function of this bypass has not been verified yet. All other genes of the methanogenesis pathway show a relatively high expression with CLR values between 0.1 and 3.4 (Supplementary Table 4), supporting a catabolic function of the encoded genes. ANME-1c encodes and expresses the methanogenesis-related membrane-bound complex H^+^-translocating F_420_:quinone oxidoreductase (*fqo*) that catalyzes the transfer of electrons from reduced cofactors to the quinone pool (Pereira et al., 2011). ANME-1c encodes an ATP synthase, which is a common feature in ANME to enable the oxidative phosphorylation of ATP, coupled to the influx of protons. ANME-1c encodes a sulfate adenylyltransferase (*cys*N; low expression, CLR = −0.02) and an adenylylsulfate kinase (*cys*C; high expression, CLR = 2.21) that could be used for assimilatory sulfate metabolism, but it lacks the key genes for dissimilatory sulfate reduction. The ANME-1c MAG lacks a complete nitrogenase operon, suggesting it is incapable of nitrogen fixation. The capability for nitrogen fixation has been shown only in ANME-2 archaea but not in ANME-1 (Dekas et al., 2009, 2015; Orphan et al., 2009; Krukenberg et al., 2018). The nitrogenase subunits *nif*DH detected in ANME-1c and other ANME-1 genomes (Meyerdierks et al., 2010) are likely paralogs of *cfb*CD because they are located in an operon with genes encoding the biosynthetic pathway of coenzyme F_430_ (Zheng et al., 2016; Moore et al., 2017). Coenzyme F_430_ functions as a prosthetic group that binds to the active site of McrA, and is therefore a key molecule for methanogens and methanotrophs (Friedmann et al., 1990; Ermler et al., 1997; Shima et al., 2012).

ANME-1c likely performs autotrophic carbon fixation *via* the carbon monoxide dehydrogenase/acetyl-CoA synthase complex (Cdh/Acs; Kellermann et al., 2012). All the *cdh* transcripts are highly abundant (CLR between 0.4 and 2.0, Supplementary Table 4), supporting the use of this pathway for autotrophy. ANME-1c does not encode other complete carbon fixation pathways. The reductive tricarboxylic acid (rTCA) cycle is incomplete, lacking the key enzyme pyruvate carboxylase. The rTCA cycle genes have relatively low expression (CLR −0.7 to 1.8, Supplementary Table 4). Enzymes of this pathway may play a role in the biosynthesis of cell building blocks (Meyerdierks et al., 2010). Like all ANME-1, ANME-1c contains a β-oxidation pathway. The phylogenetically related multi-carbon alkane oxidizers, *Ca.* Syntrophoarchaeales, *Ca.* Alkanophagales and *Ca.* Santabarbaracales harbor several copies of the β-oxidation genes and use the encoded pathway to split alkane-derived acyl-CoA into acetyl-CoA units (Laso-Pérez et al., 2016; Wang et al., 2021). However, the expression of β-oxidation genes in ANME-1c is relatively low, especially the first two reactions (CLR −0.2 to 0.5, Supplementary Table 4). Furthermore, ANME-1c lacks the electron transfer flavoprotein (*etf*AB) needed to oxidize acyl-CoA to enoyl-CoA. Hence, β-oxidation may not serve a catabolic function in ANME-1c, but play a role in biosynthesis of cell compounds. Wang and colleagues suggested that the ancestor of Syntrophoarchaeia (family including ANME-1, *Ca.* Syntrophoarchaeales and *Ca.* Alkanophagales) activated multi-carbon alkanes with their multi-carbon-alkane specific Mcr (Acrs) forming the corresponding alkyl-CoM as intermediate (Laso-Pérez et al., 2016; Wang et al., 2021). It was proposed that ANME-1 acquired a methane-activating Mcr from methylotrophic methanogens, likely from the clade *Ca.* Methanofastidiosa/*Ca.* Nuwarchaeia, and later lost the *acr* genes (Borrel et al., 2019; Wang et al., 2021).

### Phylogeny and environmental distribution of AOM-associated *Thermodesulfobacteria*

We compared the *Thermodesulfobacterium* MAG in AOM70 cultures with the *Thermodesulfobacteria* MAGs from Pescadero Basin and to MAGs retrieved from databases (NCBI and JGI). Our AOM70 *Thermodesulfobacterium* shares >95% ANI with a MAG of a *Thermodesulfobacterium* from Pescadero Basin (Laso-Pérez et al., 2022; Speth et al., 2022; Supplementary Figure 7). Based on 16S rRNA phylogeny (Supplementary Figure 6), the *Thermodesulfobacterium* AOM70 sequences form a cluster with sequences originating from Guaymas Basin and Pescadero Basin hydrothermal seeps (McKay et al., 2016; Lagostina et al., 2021; Pérez Castro et al., 2021; Speth et al., 2022). Several species of *Thermodesulfobacterium* have been isolated from hot springs (Zeikus et al., 1983; Sonne-Hansen and Ahring, 1999; Hamilton-Brehm et al., 2013), petroleum reservoirs (Rozanova and Khudiakova, 1974) and hydrothermal vents (Jeanthon et al., 2002; Moussard et al., 2004). The 16S rRNA gene sequence of our AOM70 *Thermodesulfobacterium* is 96% identical to the closest cultured representative, *Thermodesulfobacterium geofontis*, isolated from Obsidian Pool, Yellowstone National Park (Hamilton-Brehm et al., 2013). Considering an ANI <83% for distinct species and >95% for the same species (Jain et al., 2018) the *Thermodesulfobacteria* MAG from the AOM70 culture metagenome and the Pescadero MAG are a new candidate species in the genus *Thermodesulfobacterium* (Supplementary Figure 8). We propose the taxon name *Candidatus* Thermodesulfobacterium torris (torris “firebrand” referring to the thermophilic lifestyle and the formation of black aggregates in the cultures).

### Metabolism of the partner bacteria *Thermodesulfobacteria*

Members of the *Thermodesulfobacteria* family have not been previously reported as partner bacteria in AOM. All *Thermodesulfobacteria* isolates are sulfate-reducing (hyper) thermophiles with growth optima between 65°C and 90°C. They differ in the range of electron donors or carbon sources they use, which include molecular hydrogen, formate, lactate, and pyruvate (Zeikus et al., 1983; Sonne-Hansen and Ahring, 1999; Jeanthon et al., 2002; Moussard et al., 2004). Similar to other members, *Ca.* T. torris encodes a complete dissimilatory sulfate reduction pathway, including sulfate adenylyltransferase (*sat*), adenylylsulfate reductase (*apr*), and dissimilatory sulfite reductase (*dsr*). In *Ca.* T. torris this pathway is highly expressed during AOM (average CLR values between 4.0 and 6.5, Supplementary Table 4). In addition, *Ca.* T. torris contains and expresses the Dsr-associated membrane complex (*dsr*KMOP) which takes up electrons from the periplasmic cytochrome *c* pool to reduce a disulfide bond in the cytoplasmic DsrC (Pereira et al., 2011; Venceslau et al., 2014). The quinone-modifying oxidoreductase (*qmo*ABC) genes are present in an operon together with the Apr genes. In fact, the Qmo membrane complex interacts with Apr through a third unknown protein and channels electrons from the membrane ubiquinones *via* electron confurcation (Ramos et al., 2012). Both the *dsr*KMOP and the *qmo*ABC transcripts have high expression (Supplementary Table 4). Other cytoplasmic enzymes commonly associated with heterodisulfide reductases, such as the methylviologen reducing hydrogenase (Mvh/Hdr), were not found in the dataset. For energy conservation, *Ca.* T. torris uses a membrane-bound NADH:ubiquinone oxidoreductase (Nuo) and an ATP synthase (Atp). Nuo couples the reduction of NAD^+^ by reduced ubiquinones in the cytoplasmic membrane to the translocation of protons to the periplasmic space. The proton gradient generated enables oxidative phosphorylation in the ATP synthase.

The reductive acetyl-CoA pathway (Wood-Ljungdahl pathway) for carbon fixation is incomplete in the genome. *Ca.* T. torris does not encode a formate dehydrogenase (*fdh*) or a carbon monoxide dehydrogenase/acetyl-CoA complex (*cdh*/*acs*), but it encodes the enzymes catalyzing C_1_-tetrahydrofolate transformations. These reactions are necessary for several cell processes including nucleic acid biosynthesis (Ducker and Rabinowitz, 2017). Instead, *Ca.* T. torris likely fixes carbon *via* the rTCA cycle. The genome codes for an almost complete rTCA cycle, lacking a succinyl-CoA synthetase. This enzyme is likely substituted by a putative acetyl-CoA synthetase encoded in the genome and highly expressed (CLR = 3.39, locus MW689_000791). Acetyl-CoA synthetases have sequence homology with succinyl-CoA synthetases and are also active toward succinate with reduced affinity (Sánchez et al., 2000). Similarly, the thermophilic partner bacterium *Ca. Desulfofervidus auxilii* and other non-symbiotic thermophilic SRB fix carbon *via* the rTCA cycle (Schauder et al., 1987; Krukenberg et al., 2016). By contrast, meso- and psychrophilic AOM partner bacteria fix carbon using the Wood-Ljungdahl pathway (Skennerton et al., 2017).

We aimed to enrich *Ca.* T. torris by incubating aliquots of the AOM70 culture with H_2_, formate, lactate, or pyruvate as electron donors. None of the substrates resulted in immediate sulfide production (Supplementary Figure 9). Pyruvate caused sulfide production after 15 days, which likely indicates the growth of originally rare microorganisms, similar as shown for mesophilic AOM cultures (Zhu et al., 2022). These incubations suggest that *Ca.* T. torris is an obligate syntrophic bacterium that fully depends on the transfer of reducing equivalents in AOM.

### Transfer of reducing equivalents between ANME-1c and *Thermodesulfobacteria*

Because ANME have no own respiratory pathways, they need to transfer the reducing equivalents liberated during AOM to their sulfate-reducing partners. Multiple mechanisms have been proposed for syntrophic fermentation, including interspecies hydrogen transfer (Schink, 1997). A canonical syntrophy based on interspecies hydrogen transfer would require membrane-bound hydrogenases in both partners. Notably, the ANME-1c MAGs code for a complete nickel-iron hydrogenase, a feature that is rare in other ANME-1 genomes. The hydrogenase database (HydDB) annotation classifies this hydrogenase within the group 1 g of hydrogenases that are typically found in thermophilic organisms (Brock et al., 1972; Fischer et al., 1983; Huber et al., 2000; Laska et al., 2003). Yet this hydrogenase is only poorly expressed (CLR < −0.3, Supplementary Table 4). In contrast, the *Ca.* T. torris MAG lacks hydrogenases. The addition of molecular hydrogen to the culture did not stimulate sulfide production in the AOM culture, which confirms that *Ca.* T. torris cannot grow on hydrogen. Based on these observations we exclude hydrogen as electron carrier from ANME-1c toward *Ca.* T. torris. Our results confirm thermodynamic models which excluded hydrogen exchange in AOM consortia (Sørensen et al., 2001). Most other AOM partner bacteria such as SeepSRB-1a and SeepSRB2 are also obligate syntrophs and do not encode hydrogenases (Nauhaus et al., 2002; Wegener et al., 2016; Krukenberg et al., 2018). *Ca. D. auxilii*, performs DIET when growing as partner in AOM or short-chain alkane oxidation at 50°C–60°C (Wegener et al., 2015; Laso-Pérez et al., 2016; Krukenberg et al., 2018; Hahn et al., 2020), but it also shows growth on hydrogen (Krukenberg et al., 2016). It has been shown that DIET allows more efficient growth than interspecies hydrogen transfer (Summers et al., 2010).

In AOM and short-chain alkane-oxidizing consortia, cells are densely packed and the intercellular space contains cytochromes and nanowire-like structures (McGlynn et al., 2015; Wegener et al., 2015; Laso-Pérez et al., 2016; Krukenberg et al., 2018). In these mesophilic and thermophilic consortia, both partners express cytochrome and *pil*A genes (Laso-Pérez et al., 2016; Krukenberg et al., 2018). The genes *pil*A (bacterial pilin) in *Ca.* T. torris and *fla*B (archaeal flagellin) in ANME-1c show a high expression in the metatranscriptomes (CLR values of 1.8 and 3.2, respectively, Supplementary Table 4). The archaeal flagellum (archaellum) is highly similar to bacterial type IV pili (Albers and Jarrell, 2015) and might also be involved in electron transfer over longer distances. The conductivity of the archaellum from the methanogen *Methanospirillum hungatei* was demonstrated, yet its possible role in interspecies electron transfer is unclear (Walker et al., 2019). Conductive filaments that enable the transport of electrons across long distances toward extracellular electron acceptors have been widely studied in *Geobacter* (Reguera et al., 2005; Malvankar et al., 2011; Shrestha et al., 2013; Adhikari et al., 2016). Reguera et al. (2005) showed that pilus-deficient *Geobacter* mutants could not transfer electrons to extracellular electron acceptors, suggesting an involvement of pilin proteins in this process (Reguera et al., 2005).

**Figure 4:**
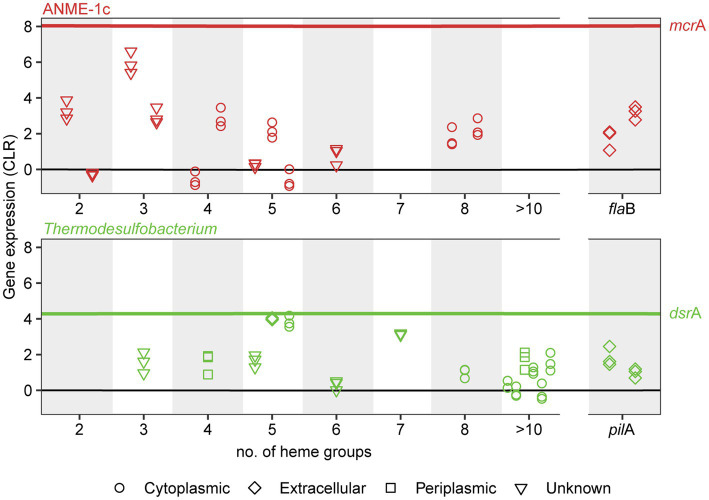
Expression and subcellular localization of multiheme cytochromes and cellular appendages in ANME-1c (top) and *Thermodesulfobacterium* (bottom) in AOM70 cultures. Gene expression is noted as centered-log ratio values, with 0 as the mean expression of all genes in each genome. *mcr*A and *dsr*A mean CLR values are displayed as a reference (red and green lines, respectively). Symbols show the predicted subcellular localization of cytochromes (PSORTb). Three symbols in a vertical line correspond to CLR values of a specific locus in triplicate metatranscriptomes.

The molecular basis of DIET in sulfate-dependent AOM has been intensively discussed in the past years (McGlynn et al., 2015; Wegener et al., 2015; Chadwick et al., 2022; Yu et al., 2022). McGlynn et al. (2015) proposed a model based on direct interspecies electron transfer *via* multiheme cytochromes for AOM consortia, using evidence from single-cell activities, microscopic observations and genomics (McGlynn et al., 2015). Krukenberg et al. (2018) showed that both partners highly express cytochromes with a low number of heme groups (3–5 heme binding motifs) during thermophilic AOM (Krukenberg et al., 2018). In AOM consortia at 60°C, it was observed that SRB *Ca. Desulfofervidus auxilii* expressed pili genes and that the intercellular space was filled with nanowire structures similar to syntrophic consortia of *Geobacter* (Wegener et al., 2015). Indeed it was recently shown that the filaments in *Geobacter sulfurreducens* are not formed by pilin proteins (PilA), but rather by stacked OmcS hexaheme cytochromes (Wang et al., 2019). Instead, PilA might be involved in secretion of OmcS cytochromes (Gu et al., 2021). Both ANME-1c and *Ca.* T. torris encode several multiheme cytochromes. ANME-1c codes for several proteins with 2 to 8 heme-binding motifs (Figure 4; Supplementary Table 4). A cytochrome c7 and a protein without annotation, both with 3 heme groups, are among the top expressed genes (CLR values of 5.9 and 2.96, respectively, Supplementary Table 4). However, the predicted subcellular localizations of these putative cytochromes are unknown, as reported by PSORTb. Interestingly, these cytochromes are highly similar to extracellular cytochromes that were highly expressed in ANME-1 during AOM at 60°C (Supplementary Table 4; Krukenberg et al., 2018). *Ca.* T. torris also contains numerous multiheme cytochromes, with up to 26 heme-binding motifs. A cytochrome-like gene with five heme-binding motifs and predicted extracellular localization shows high expression levels similar to the *dsr*A (CLR value of 4.0, Figure 4). This putative pentaheme cytochrome shares high sequence identity (<40% identity) with a *Ca. Desulfofervidus auxilii* OmcS-like protein (Wegener et al., 2015; Krukenberg et al., 2018) (locus tag HS1_000170, Supplementary Figure 10). We hypothesize that the extracellular pentaheme cytochromes from *Ca.* T. torris are likely involved in receiving electrons derived from methane oxidation. ANME-1c cytochromes with undetermined cell localization might also be involved in interspecies electron transfer.

### Alternative roles of the membrane-bound hydrogenase in ANME-1c

Some ANME-1c MAGs code for a NiFe membrane-bound hydrogenase, which is an uncommon feature in most ANME genomes (Stokke et al., 2012; Wegener et al., 2015; Krukenberg et al., 2018). This hydrogenase forms a clade with those of *Ca.* Syntrophoarchaeum and *Ca.* Alkanophagales (Figure 2B; Supplementary Figure 11; Laso-Pérez et al., 2016; Wang et al., 2021). In *Ca.* Syntrophoarchaeum, the hydrogenase is highly expressed during anaerobic propane and butane oxidation, albeit its function is also unknown (Laso-Pérez et al., 2016). In contrast, in ANME-1c the NiFe-hydrogenase has low expression (Figure 3; Supplementary Table 4). A recent study described the ANME-1c as an ancestral clade at the base of ANME-1a/1b and as a sister branch of *Ca.* Syntrophoarchaeales (Laso-Pérez et al., 2022). ANME-1c were proposed to be facultative methanogens based on the encoded hydrogenase and the unclear association with partner bacteria in environmental samples (Laso-Pérez et al., 2022). The capability of ANME-1 to perform methanogenesis has been repeatedly suggested (Seifert et al., 2006; Treude et al., 2007; Orcutt et al., 2008). ANME-1 16S rRNA and *mcr*A genes and transcripts were found in methanogenic sediment horizons at White Oak River estuary and in the sulfate–methane transition zone (Lloyd et al., 2011; Kevorkian et al., 2021). The authors weighted this as an argument for a potential role of ANME-1 in methanogenesis. There is no genomic evidence that ANME-1 from these environment contain hydrogenases, which are required to perform methanogenesis from CO_2_, and this question should be addressed in future metagenomic studies. To test whether ANME-1c are capable of methanogenesis, we transferred AOM70 culture aliquots to sulfate-free medium and exchanged the methane in the headspace with an H_2_/CO_2_ atmosphere. Hydrogen addition did not stimulate production of methane in AOM70 cultures in the course of 4-month incubations (Supplementary Figure 12). According to these results, ANME-1c are incapable of hydrogenotrophic methanogenesis. The habitable zones of the hydrothermally-heated sediments in Guaymas Basin are rich in sulfate due to hydrothermal circulation and seawater advection (Ramírez et al., 2021). This provides additional evidence for ANME-1c being obligate methane oxidizers that depend on syntrophic partnerships with sulfate-reducers. The hydrogenase in ANME-1c might be a remnant from the common ancestor of the *Ca.* Syntrophoarchaeum/*Ca.* Alkanophagales/ANME-1 clade. These ancestral alkanotrophic archaea might have performed interspecies electron transfer based on hydrogen transfer. In the course of evolution that capability was replaced by an apparently more efficient DIET mechanism *via* extracellular multiheme cytochromes (Summers et al., 2010).

## Conclusion

Here we cultured a thermophilic AOM consortium at 70°C consisting of methane-oxidizing archaea from the ANME-1c clade with *Thermodesulfobacteria* as sulfate-reducing partner bacteria. Our study bridges the temperature gap between AOM activity previously observed by pore water profiles and tracer experiments, and its *in vitro* demonstration in cultures. ANME-1c is a basal lineage to the ANME-1a/b clade. Interestingly, ANME-1c MAGs encode a hydrogenase operon that is not present in the ANME-1a/b clades. ANME-1c neither produces nor consumes hydrogen. The hydrogenase genes have low expression and this enzyme is likely a remnant of the ancestor of the *Ca.* Syntrophoarchaeia, an organism that was likely a multi-carbon alkane oxidizer. The function of this hydrogenase in ANME-1, but also other members of the Syntrophoarchaeia is unresolved. Based on indirect evidence ANME-1 were repeatedly suggested to be facultative methanogens (Seifert et al., 2006; Treude et al., 2007; Orcutt et al., 2008; Lloyd et al., 2011; Kevorkian et al., 2021). Here, we demonstrated that even ANME-1c that encode a hydrogenase are not able to reverse their metabolism toward net methanogenesis. Cultivation-based approaches should be used to test whether the ANME-1a/b that encode hydrogenases are capable of methanogenesis.

Most likely, ANME and their partners interact *via* DIET. The partner *Thermodesulfobacterium* encodes an extracellular pentaheme c-type cytochrome with high expression. This cytochrome is highly similar to *Ca. Desulfofervidus auxilii* cytochromes that have high expression during AOM at 60°C. This evidence suggests a central role of this pentaheme cytochrome of *Thermodesulfobacteria* in DIET. In addition, multiheme cytochromes and flagella from ANME-1c are highly expressed under AOM conditions. However, the role of these cytochromes with unknown subcellular location in DIET needs further investigation.

Our study provides culture-based evidence for the feasibility of AOM at high-temperature conditions and the versatility and evolutionary diversity of the organisms mediating AOM in marine environments. To our knowledge, this is the first report of syntrophic consortia of ANME and *Thermodesulfobacteria.* ANME-1c and *Ca.* T. torris co-occur in environmental samples from the Guaymas Basin and Pescadero Basin (Gulf of California; Laso-Pérez et al., 2022; Speth et al., 2022). This limited geographical distribution is likely a result of undersampling of heated methane-rich environments. Apart from the rather rare methane-rich hydrothermal vents, consortia of these phylotypes might inhabit deep sulfate–methane interfaces. Indeed, these sulfate–methane interfaces occur at depths up to 150 m below the seafloor and at temperatures of up to 80°C (Teske et al., 2021; Beulig et al., 2022). The question of whether these sulfate–methane interfaces are a habitat for AOM needs to be addressed. Future studies should aim to search for the ANME-1c and *Thermodesulfobacteria* co-occurring in such deep-sea sediments.

## Data availability statement

The metagenome-assembled genomes (MAGs) and metagenomic contigs are available on NCBI under BioProject PRJNA805391. Assembled metagenomic contigs are available under BioSample ID SAMN30121676. ANME-1c and Ca. Thermodesulfobacterium torris annotated genomes are available under BioSample IDs SAMN27514932 and SAMN27514933, respectively.

## Author contributions

DBM and GW designed the study. GW did sampling on board. AT planned and organized the cruise. DBM and HZ did cultivation experiments. DBM performed laboratory experiments and ‘omics analyses and wrote the manuscript with contributions from all coauthors. All authors contributed to the article and approved the submitted version.

## Funding

The study was funded by the Max Planck Society and the DFG under Germany’s Excellence Initiative/Strategy through the Clusters of Excellence EXC 2077 “The Ocean Floor—Earth’s Uncharted Interface” (project no. 390741601). The Guaymas Basin expedition was supported by the National Science Foundation, Biological Oceanography grant no. 1357238 to AT (Collaborative Research: Microbial Carbon cycling and its interactions with Sulfur and Nitrogen transformations in Guaymas Basin hydrothermal sediments).

## Conflict of interest

The authors declare that the research was conducted in the absence of any commercial or financial relationships that could be construed as a potential conflict of interest.

The handling editor SER declared past co-authorships with one of the authors GW.

## Publisher’s note

All claims expressed in this article are solely those of the authors and do not necessarily represent those of their affiliated organizations, or those of the publisher, the editors and the reviewers. Any product that may be evaluated in this article, or claim that may be made by its manufacturer, is not guaranteed or endorsed by the publisher.

## Footnotes

^1^ https://sourceforge.net/projects/bbmap/

^2^ https://github.com/dbenitom/Metagenomics_scripts/blob/main/CXXCH_search_anvio_import.sh

## Supplementary material

SUPPLEMENTARY FIGURE 1 Sulfide production in initial sediment slurry and in AOM cultures at 70°C.

SUPPLEMENTARY FIGURE 2 16S rRNA community composition of original sediments.

SUPPLEMENTARY FIGURE 3 CARD-FISH micrographs of AOM the culture at 70°C.

SUPPLEMENTARY FIGURE 4 16S rRNA phylogeny of ANME-1, including ANME-1c.

SUPPLEMENTARY FIGURE 5 Average nucleotide identity of ANME-1c genomes.

SUPPLEMENTARY FIGURE 6 16S rRNA phylogeny of *Thermodesulfobacteria*.

SUPPLEMENTARY FIGURE 7 Phylogenomic tree of *Thermodesulfobacteria*.

SUPPLEMENTARY FIGURE 8 Average nucleotide identity of selected *Thermodesulfobacteria* genomes.

SUPPLEMENTARY FIGURE 9 Sulfide production in incubations with substrates for *Thermodesulfobacteria*.

SUPPLEMENTARY FIGURE 10 Alignment of Ca. Thermodesulfobacterium torris and partner sulfate-reducing bacteria multiheme cytochromes likely involved in interspecies electron transfer.

SUPPLEMENTARY FIGURE 11 NiFe hydrogenase tree including ANME-1 hydrogenases.

SUPPLEMENTARY FIGURE 12 The AOM70 enrichment does not produce methane under methanogenesis conditions.

SUPPLEMENTARY TABLE 1 Abundance and length distribution of PacBio metagenomic long reads.

SUPPLEMENTARY TABLE 2 CARD-FISH probes used in this study.

SUPPLEMENTARY TABLE 3 Genomes and conserved marker genes used for archaeal/bacterial phylogenomic tree calculation.

SUPPLEMENTARY TABLE 4 Annotation and metatranscriptomic expression of main pathways of ANME-1c and Ca. Thermodesulfobacterium torris discussed in the main text.

SUPPLEMENTARY TABLE 5 Reference hydrogenase accession numbers used for hydrogenase phylogenetic tree calculation.
