# Supplementary material for: Deep-branching ANME-1c archaea grow at the upper temperature limit of anaerobic oxidation of methane
Source: Front Microbiol. 2022 Sep 23;13:988871. doi: 10.3389/fmicb.2022.988871 (PMC9539880; doi:10.3389/fmicb.2022.988871)
Supplement: SUPPLEMENTARY FIGURE 1 — Sulfide production in initial sediment slurry and in AOM cultures at 70°C. [file Data_Sheet_1.pdf]

Deep-branching ANME-1c archaea grow at the upper temperature limit of anaerobic oxidation of methane

David Benito Merino[1,2], Hanna Zehnle[1,2,3], Andreas Teske[4], Gunter Wegener[1,3*]

[1]Max Planck Institute for Marine Microbiology, Bremen, Germany

[2]Faculty of Geosciences, University of Bremen, Bremen, Germany

[3]MARUM, Center for Marine Environmental Sciences, University of Bremen, Bremen, Germany

[4]Department of Marine Sciences, University of North Carolina at Chapel Hill, Chapel Hill, NC, USA

*** Correspondence:**
David Benito Merino, dbenito@mpi-bremen.de

Gunter Wegener, gwegener@mpi-bremen.de

Supplementary Material

1 Supplementary Figures

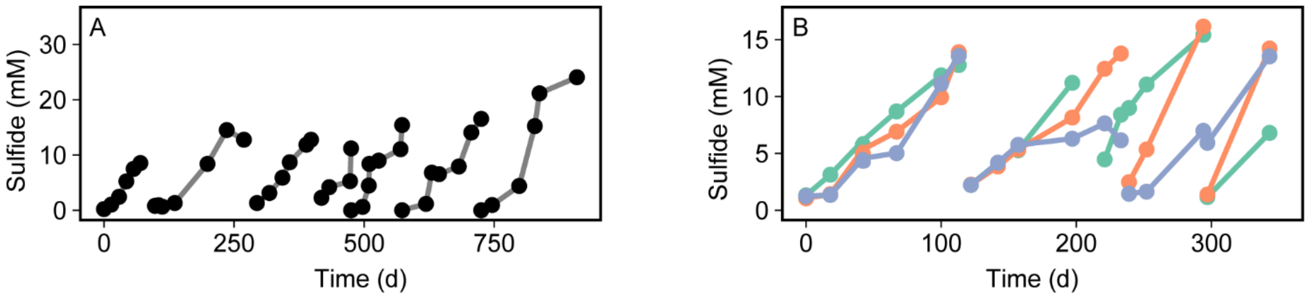

Supplementary Figure 1. Sulfide production in initial sediment slurry and in AOM cultures at 70°C. A. Sulfide production from sediment slurry was monitored over 800 days. Every drop in sulfide levels indicates when the slurry was diluted 1/10 with fresh medium. **B.** Sulfide production from three AOM70 cultures over 400 days. Every drop in sulfide levels indicates when the medium was replaced with fresh sulfate-reducer medium (no dilutions were made in established cultures).

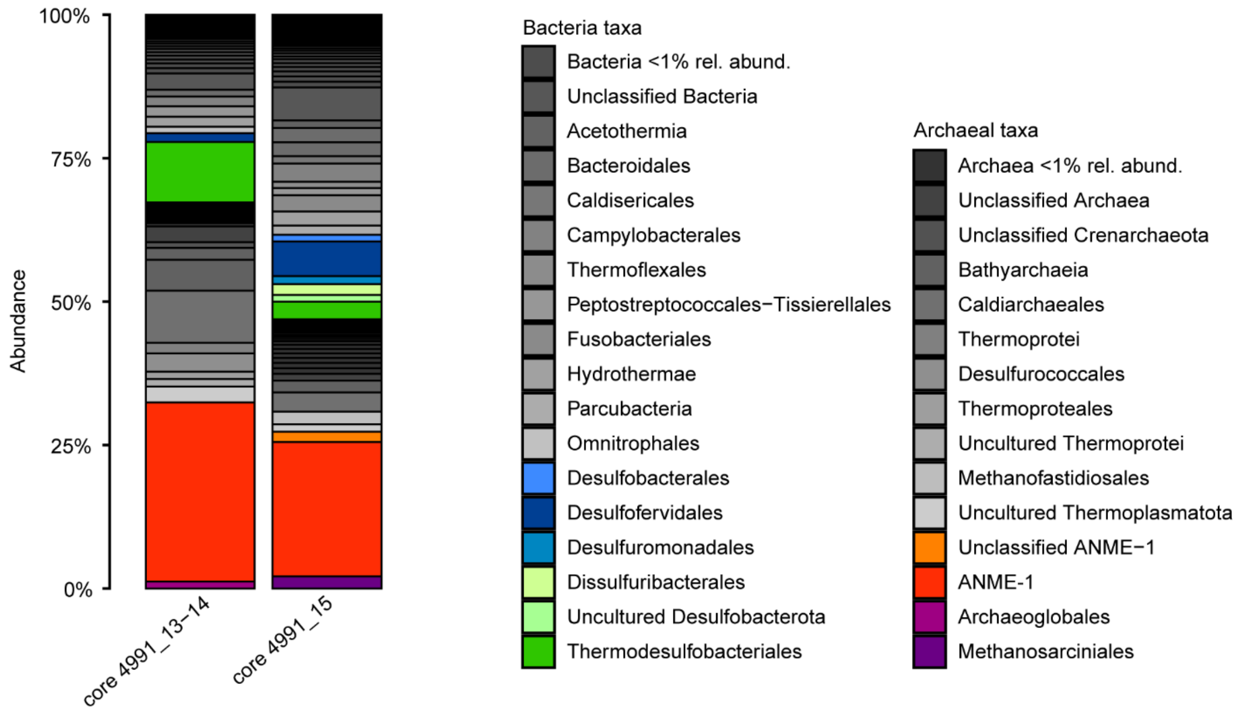

Supplementary Figure 2. 16S rRNA community composition of original sediments. Data from two sediment core metagenomes are shown (core 4991_13-14 and core 4991_15). The 16S rRNA gene reads were extracted, mapped and classified with phyloFlash (Gruber-Vodicka et al., 2020). Bacterial sulfate reducers of the Desulfobacterota phylum and the archaeal phylum Halobacterota are highlighted in colours. *Thermodesulfobacteria* were found in both sediment cores. Unclassified ANME-1 (ANME-1c) were only found in one core with relative abundances over 1%. The ANME-1c and *Thermodesulfobacteria* 16S rRNA sequences reconstructed from sediment core metagenomes are also shown in the phylogenetic tree in Supplementary Figure 4 and Supplementary Figure 6, respectively.

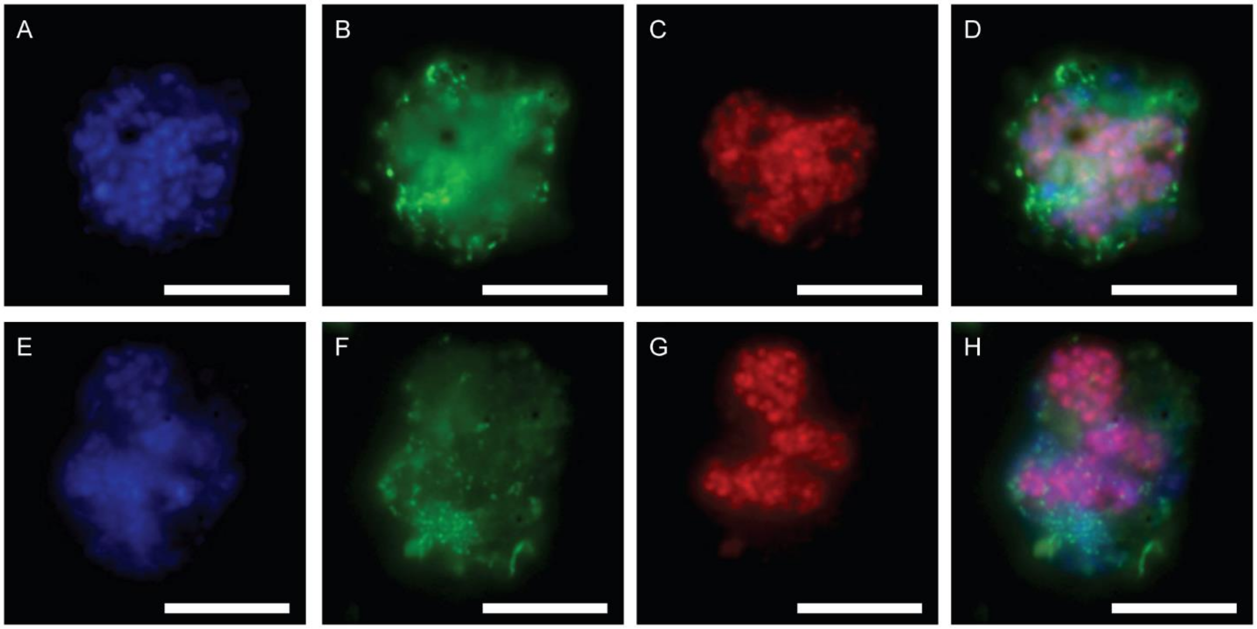

Supplementary Figure 3. CARD-FISH micrographs of the AOM culture at 70°C. Micrographs of two different aggregates of ANME-1c and *Ca.* Thermodesulfobacterium torris. A, E: DAPI staining; B, F: general bacterial probe mix (EUB338 I-III): C, G: ANME-1c specific probe (ANME-1-389); D, H: merged images of DAPI, EUB338 and ANME-1-389. Scale bar: 10 µm.

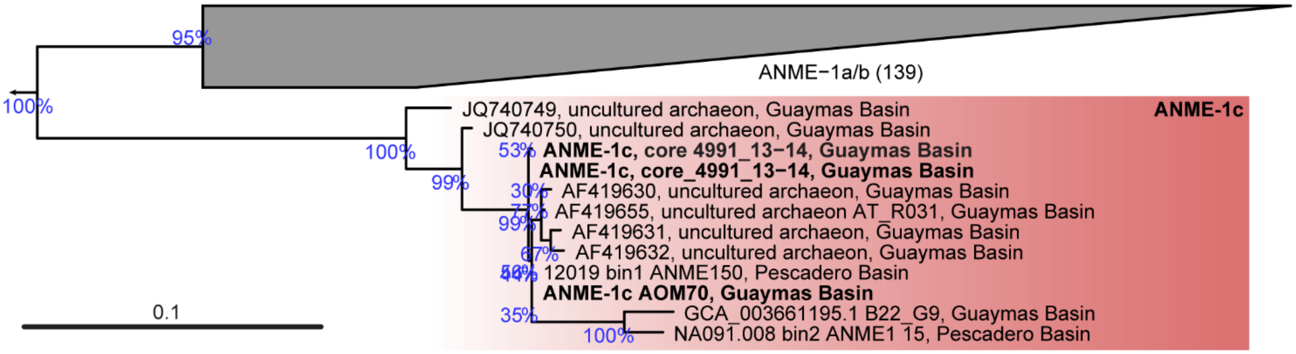

Supplementary Figure 4. 16S rRNA phylogeny of ANME-1c. Maximum likelihood tree of selected ANME-1 16S rRNA gene sequences from SILVA release 138.1 and reference NCBI genomes. *Methanonatronarchaea* sequences served as outgroup (not shown). Blue numbers at branching points shown support values based on 1000 bootstraps. ANME-1c 16S rRNA genes (red shading) form a basal clade to ANME-1a/b. The 16S rRNA gene sequences of ANME-1c from AOM70 culture and sediment samples appear in bold.

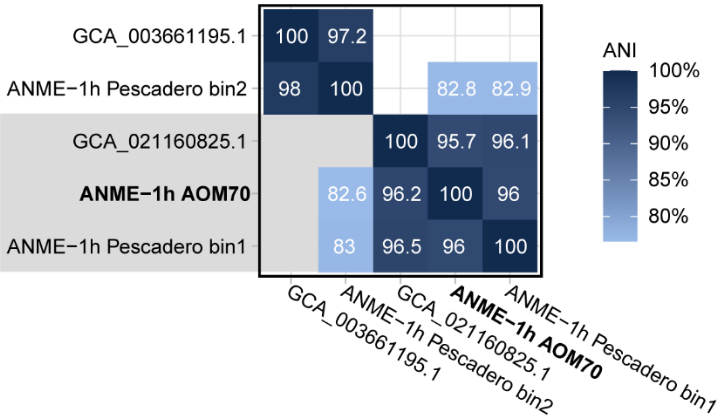

Supplementary Figure 5. Average nucleotide identity of ANME-1c genomes. The ANME-1c clade includes 5 MAGs distributed in 2 species (>95% ANI, grey shading). ANME-1c from AOM70 enrichment (this study) belongs to the same species as another MAG from the Guaymas Basin.

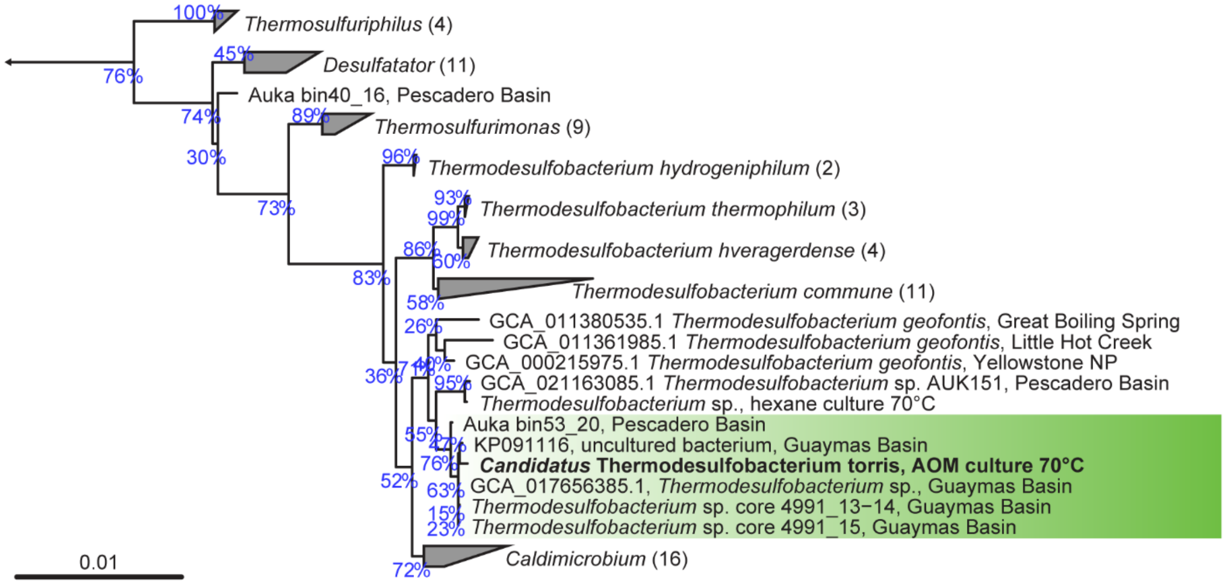

Supplementary Figure 6. 16S rRNA gene phylogeny of *Thermodesulfobacteria*. Maximum likelihood tree of selected *Thermodesulfobacteria* 16S rRNA gene sequences from SILVA 138.1 and reference NCBI genomes. *Ca.* Desulfofervidus sequences served as outgroup (not shown). Blue numbers at branching points shown support values based on 1000 bootstraps. The 16S rRNA gene sequences of *Thermodesulfobacteria* from AOM70 culture and sediment samples appear in bold. The *Ca.* Thermodesulfobacterium torris clade (green shading) contains sequences with similarities >99%. *Ca.* T. torris 16S rRNA is 97% identical to the closest cultured representative, *Thermodesulfobacterium geofontis*.

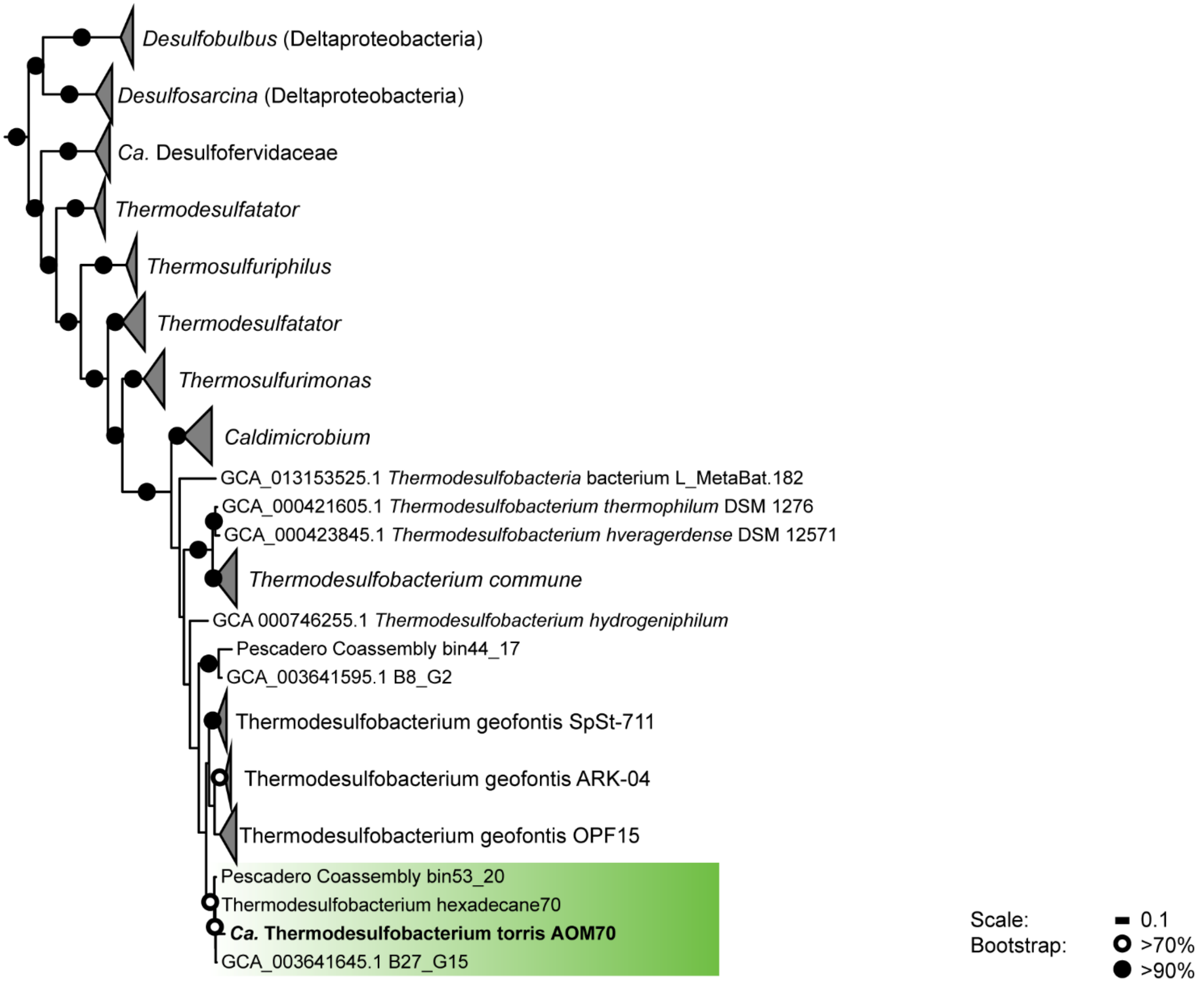

Supplementary Figure 7. Phylogenomic tree of *Thermodesulfobacteria*. Maximum likelihood tree based on a concatenated alignment of 71 bacterial marker genes (Rinke et al., 2013) (Supplementary Table 2). Bootstrap support is based on 500 iterations.

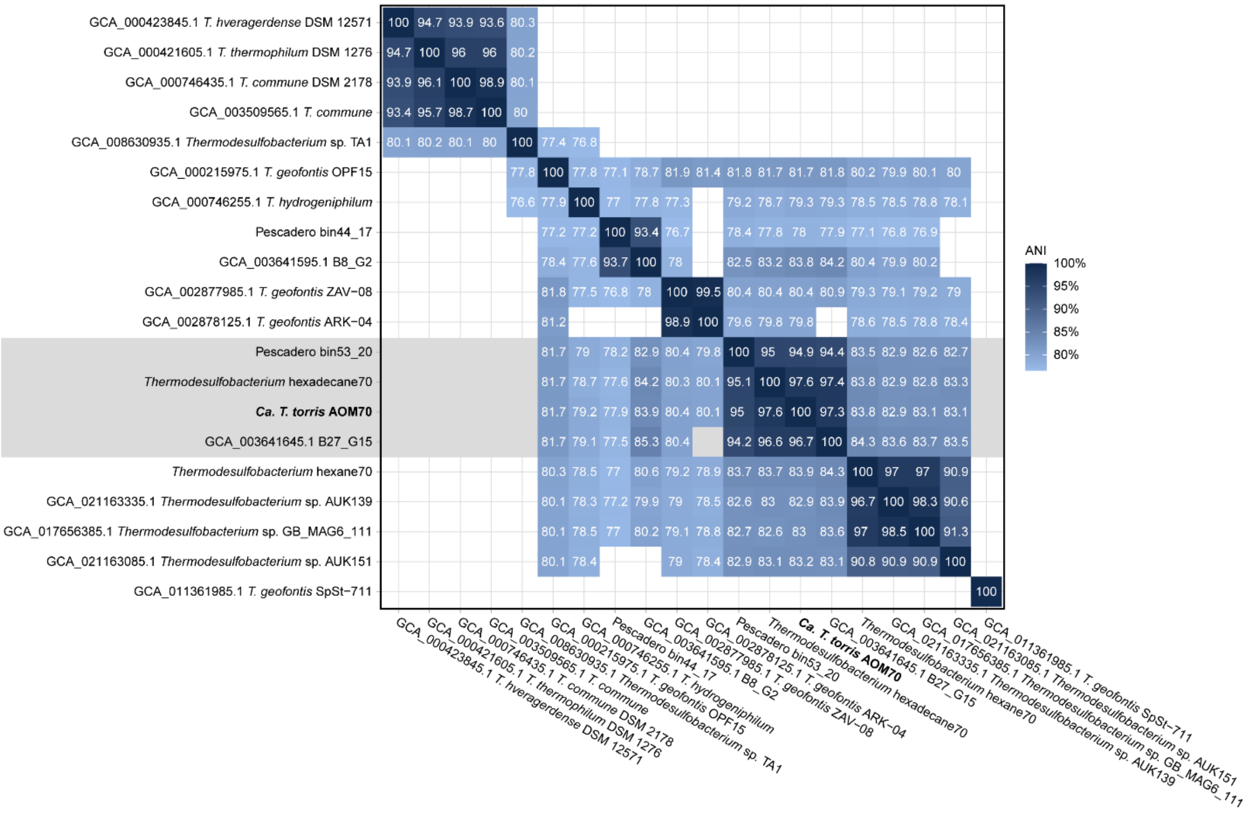

Supplementary Figure 8. Average nucleotide identity of selected *Thermodesulfobacteria* genomes. *Ca.* Thermodesulfobacterium torris (in bold) species cluster (>95% ANI, grey shading) includes 4 genomes: two MAGs originating from Pescadero Basin (Laso-Pérez et al., 2022; Speth et al., 2022), a MAG from a hexadecane-degrading enrichment at 70 °C from Guaymas Basin (Benito Merino, unpublished) and the MAG from the AOM70 enrichment from Guaymas Basin (this study).

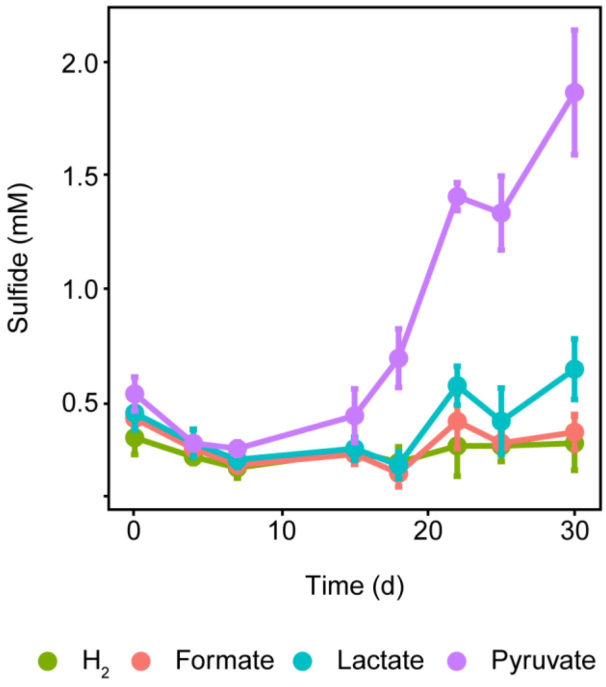

Supplementary Figure 9. Sulfide production in incubations with substrates for *Thermodesulfobacteria*. AOM70 culture aliquots were incubated with formate (10 mM), lactate (10 mM), pyruvate (10 mM) or hydrogen (2 atm H_2:CO_2 20:80 headspace). Substantial substrate-dependent sulfide oxidation was only observed in incubations with pyruvate after 15 days, most likely caused by ancillary heterotrophic microorganisms and not by *Thermodesulfobacteria*.

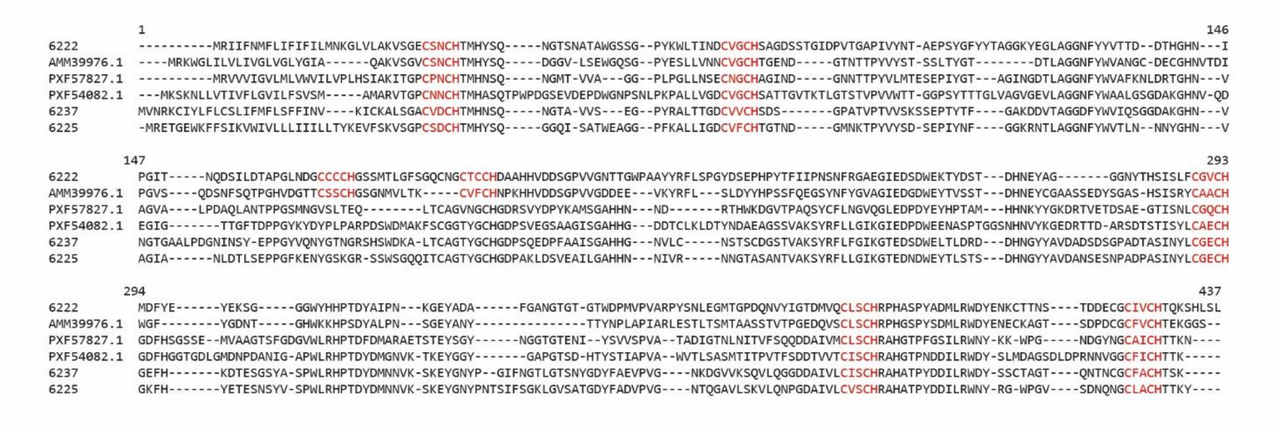

Supplementary Figure 10. Alignment of *Ca*. Thermodesulfobacterium torris and partner sulfate-reducing bacteria multiheme cytochromes likely involved in interspecies electron transfer. *Ca*. T. torris cytochromes: 6237 (locus tag MW689_000279, extracellular, 5 heme), 6222 (locus tag MW689_000264, unknown location, 7 heme), 6225 (locus tag MW689_000267, unknown location, 5 heme). *Ca*. D. auxilii cytochrome: AMM39976.1 (locus tag HS1_000170). Seep-SRB2 (E20 culture) cytochrome: PXF57827.1 (locus tag C4B58_08455). Seep-SRB2 (G37 culture) cytochrome: PXF54082.1 (locus tag: C4B57_08635). Putative heme-binding motifs (CxxCH) highlighted in red have conserved positions within the alignment.

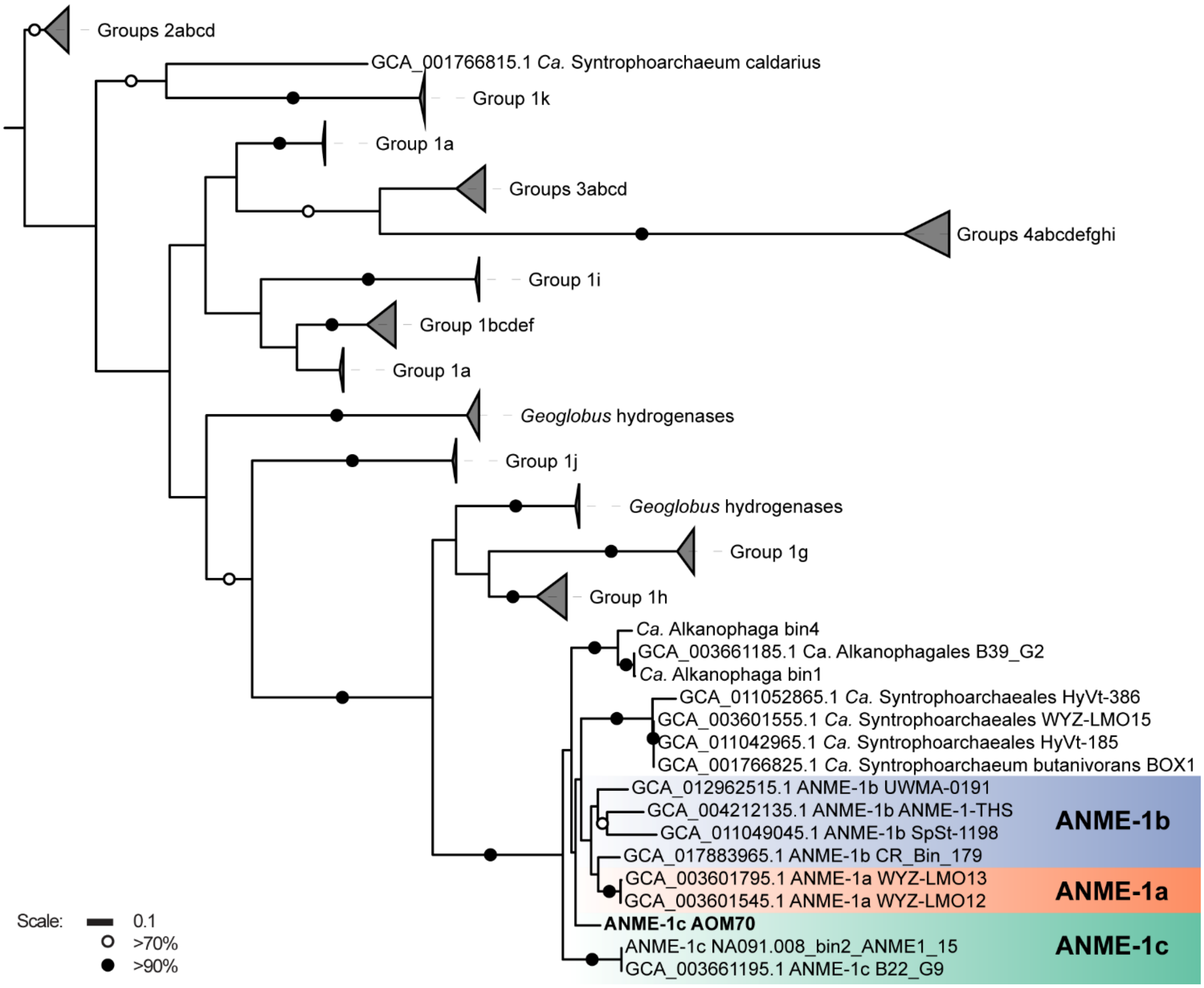

Supplementary Figure 11. NiFe hydrogenase tree including ANME-1 hydrogenases. All ANME-1 and related Syntrophoarchaeales and Alkanophagales hydrogenases form a cluster at the base of groups 1g and 1h NiFe hydrogenases. The low bootstrap support (>70%) explains the spread of the hydrogenases of the ANME-1c.

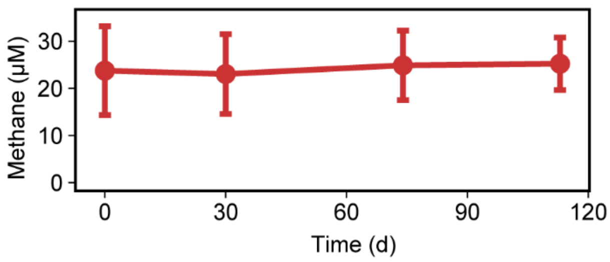

Supplementary Figure 12. The AOM70 enrichment does not produce methane under methanogenesis conditions. Triplicate aliquots of AOM70 cultures were transferred to sulfate-free medium with an H_2/CO_2 headspace. We did not observe methane formation over time. The ~24 µM methane in the headspace was residual methane from the original AOM70 culture.

2 Supplementary Tables

Supplementary Table 1. Abundance and length distribution of PacBio metagenomic long reads.

Read length	Abundance	Total length (bp)
>100 bp	15,363	143,242,211
>250 bp	15,154	143,199,554
>500 bp	14,335	142,905,803
>1 Kbp	13,602	142,378,411
>2.5 Kbp	11,677	139,017,257
>5 Kbp	9,104	129,533,143
>10 Kbp	5,899	106,112,415
>25 Kbp	817	24,768,328
>50 Kbp	4	204,380

frontiers

Supplementary Table 2. CARD-FISH probes used in this study.

**Probe**	**Target group**	**[FA]**	**Probe sequence (5' to 3')**	**Reference**	**Hybridisation**
Arch915	Most archaea	35%	GTG CTC CCC CGC CAA TTC CT	(Amann et al., 1990)	Hybridized with ANME-1c.
EUB338 I-III	Most bacteria	35%	I: GCT GCC TCC CGT AGG AGT<br>II: GCA GCC ACC CGT AGG TGT<br>III: GCT GCC ACC CGT AGG TGT	(Daims et al., 1999)	Hybridized with SRB.
ANME-1-350	ANME-1	40%	AGT TTT CGC GCC TGA TGC	(Boetius et al., 2000)	Did not hybridize with ANME-1c.
ANME-1-260	ANME-1c	30%	GGC TTG TCG GTC CGT TAA	This study.	Hybridized with ANME-1c.
ANME-1-389	ANME-1c	40%	CAC TCA GCG TCC CCT CAT	This study.	Hybridized with ANME-1c.
Tds201	Thermodesulfo-bacteria	-	GCT TGC TAT GCA GAG GCC	This study.	No hybridization
Tds725	Thermodesulfo-bacteria	-	CCA GCT GGC CGG CTT CCC	This study.	No hybridization
Tds991	Thermodesulfo-bacteria	-	ACA ACC CTG GCA TGT CAA	This study.	No hybridization

Supplementary Table 3. Genomes and conserved marker genes used for archaeal/bacterial phylogenomic tree calculation (see attached spreadsheet).

Supplementary Table 4. Annotation and metatranscriptomic expression of main pathways of ANME-1c and *Ca.* Thermodesulfobacterium torris discussed in the main text (see attached spreadsheet).

Supplementary Table 5. Reference hydrogenase accession numbers used for hydrogenase phylogenetic tree calculation (see attached spreadsheet).

3 References

Amann, R. I., Krumholz, L., and Stahl, D. A. (1990). Fluorescent-oligonucleotide probing of whole cells for determinative, phylogenetic, and environmental studies in microbiology. *J. Bacteriol.* 172, 762. doi: 10.1128/JB.172.2.762-770.1990.

Boetius, A., Ravenschlag, K., Schubert, C. J., Rickert[2], D., Widdel, F., Gieseke, A., et al. (2000). A marine microbial consortium apparently mediating anaerobic oxidation of methane. *Nature* 407, 623–626.

Daims, H., Brühl, A., Amann, R., Schleifer, K.-H., and Wagner, M. (1999). The domain-specific probe EUB338 is insufficient for the detection of all Bacteria: Development and evaluation of a more comprehensive probe set. *Syst. Appl. Microbiol.* 22, 434–444. doi: 10.1016/S0723-2020(99)80053-8.

Gruber-Vodicka, H. R., Seah, B. K. B., and Pruesse, E. (2020). phyloFlash: Rapid small-subunit rRNA profiling and targeted assembly from metagenomes. *mSystems* 5. doi: 10.1128/mSystems.00920-20.

Laso-Pérez, R., Wu, F., Crémière, A., Speth, D. R., Magyar, J. S., Krupovic, M., et al. (2022). Evolutionary diversification of methanotrophic Ca. Methanophagales (ANME-1) and their expansive virome. *bioRxiv*, 2022.07.04.498658. doi: 10.1101/2022.07.04.498658.

Rinke, C., Schwientek, P., Sczyrba, A., Ivanova, N. N., Anderson, I. J., Cheng, J. F., et al. (2013). Insights into the phylogeny and coding potential of microbial dark matter. *Nat. 2013 4997459* 499, 431–437. doi: 10.1038/nature12352.

Speth, D. R., Yu, F. B., Connon, S. A., Lim, S., Magyar, J. S., Peña-Salinas, M. E., et al. (2022). Microbial communities of Auka hydrothermal sediments shed light on vent biogeography and the evolutionary history of thermophily. *ISME J. 2022*, 1–15. doi: 10.1038/s41396-022-01222-x.
